# Caring for a Dying Patient; a Multidisciplinary Team Survey Across the Acute Old Age Psychiatry Inpatient Wards at the Royal Edinburgh Hospital, NHS Lothian, Scotland

**DOI:** 10.1192/bjo.2026.11550

**Published:** 2026-06-30

**Authors:** Catherine-Anne Convery, Carol-Anne Sherriff, Carrie Coull

**Affiliations:** Royal Edinburgh Hospital, NHS Lothian, Edinburgh, United Kingdom

## Abstract

**Aims:**

The psychiatric specialty of Old Age Psychiatry cares for a patient population with complex psychiatric needs, where complex medical comorbidity and increasing frailty are common. As a result, patients may die while under inpatient psychiatric care. This survey aimed to gather views from the Multidisciplinary Team (MDT) working across the Acute Old Age Psychiatry wards at the Royal Edinburgh Hospital (REH) on their experience of providing care for a dying patient. Between January 2023 and December 2024, 14 patients were cared for and died of medical causes on the inpatient wards.

**Methods:**

In January 2025, a Microsoft Form survey asking 3 questions was constructed regarding MDT involvement in the care of a dying patient;

What do you feel is done well?

What challenges do you encounter?

Regarding challenges encountered, how do you think they could be improved/overcome/better supported?

The survey was emailed to all MDT members in the Acute Old Age Psychiatry inpatient serviceatREH. The qualitative responses were gathered and collated into a ‘word cloud’.

**Results:**

16 survey responses from the MDT were returned; 5 Nurses, 4 Nursing Assistants, 3 Doctors, 3 Arts Psychotherapists and 1 Pharmacist.

Regarding the aspects of care for a dying patient which were done well, the MDT highlighted; patient-centered care, proactive symptom management, patients are treated with compassion, gentleness, dignity and respect. Furthermore, collaborative MDT working, involvement of family and spiritual care and promoting a calm environment were noted.

Challenges encountered by the MDT mainly focused on logistical difficulties for initiating and managing administration of palliative medications via syringe driver in the psychiatric setting. At times, additional support from the local Community District Nursing team is required to deliver this aspect of patient care. Other difficulties reflected the challenge personally to staff in processing a patient death and considering howbest to support other patients following a death.

Recommendations from the MDT to address these challenges identified opportunities for education and training, called for additional staffing and consideration of informal MDT support check ins.

**Conclusion:**

This survey highlights important views from the MDT on the experience of caring for a dying patient. In response, additional nursing staff have been trained in the implementation and management of syringe drivers to administer palliative medications and amodule inpalliative care has been added to the newly qualified nurses training programme for the Acute Old Age Psychiatry wards at REH.

